# Collateral-resistance to estrogen and HER-activated growth is associated with modified AKT, ERα, and cell-cycle signaling in a breast cancer model

**DOI:** 10.37349/etat.2022.00074

**Published:** 2022-02-28

**Authors:** Kate M. Moore, Vera Cerqueira, Kenneth G. MacLeod, Peter Mullen, Richard L. Hayward, Simon Green, David J. Harrison, David A. Cameron, Simon P. Langdon

**Affiliations:** 1 Cancer Research UK Edinburgh Centre, Institute of Genetics and Cancer, University of Edinburgh, Crewe Road South, EH4 2XR Edinburgh, UK; 2 Cancer Research UK Barts Centre, Barts and The London School of Medicine and Dentistry, Queen Mary University of London, Charterhouse Square, EC1M 6BQ London, UK; 3 West of Scotland Clinical Genetics Service, Queen Elizabeth University Hospital, G51 4TF Glasgow, UK; 4 School of Medicine, University of St Andrews, North Haugh, KY16 9TF St Andrews, UK; 5 Cyclacel Ltd, James Lindsay Place, Dundee Technopole, DD1 5JJ Dundee, UK; University of Aberdeen, UK

**Keywords:** Breast cancer, endocrine resistance, estrogen, erbB receptor, seliciclib

## Abstract

**Aim:**

A model of progressively endocrine-resistant breast cancer was investigated to identify changes that can occur in signaling pathways after endocrine manipulation.

**Methods:**

The MCF7 breast cancer model is sensitive to estrogens and anti-estrogens while variant lines previously derived from wild-type MCF7 are either relatively 17β-estradiol (E_2 _)-insensitive (LCC1) or fully resistant to estrogen and anti-estrogens (LCC9).

**Results:**

In LCC1 and LCC9 cell lines, loss of estrogen sensitivity was accompanied by loss of growth response to transforming growth factor alpha (TGFα), heregulin-beta and pertuzumab. LCC1 and LCC9 cells had enhanced AKT phosphorylation relative to MCF7 which was reflected in downstream activation of phospho-mechanistic target of rapamycin (mTOR), phospho-S6, and phospho-estrogen receptor alpha Ser167 [ERα(Ser167)]. Both AKT2 and AKT3 were phosphorylated in the resistant cell lines, but small interfering RNA (siRNA) knockdown suggested that all three AKT isoforms contributed to growth response. ERα(Ser118) phosphorylation was increased by E_2_ and TGFα in MCF7, by E_2_ only in LCC1, but by neither in LCC9 cells. Multiple alterations in E_2_-mediated cell cycle control were identified in the endocrine-resistant cell lines including increased expression of *MYC*, *cyclin A1*, *cyclin D1*, cyclin-dependent kinase 1 (*CDK1*), *CDK2*, and hyperphosphorylated retinoblastoma protein (ppRb), whereas p21 and p27 were reduced. Estrogen modulated expression of these regulators in MCF7 and LCC1 cells but not in LCC9 cells. Seliciclib inhibited CDK2 activation in MCF7 cells but not in resistant variants; in all lines, it reduced ppRb, increased p53 associated responses including p21, p53 up-regulated modulator of apoptosis (PUMA), and p53 apoptosis-inducing protein 1 (p53AIP1), inhibited growth, and produced G2/M block and apoptosis.

**Conclusions:**

Multiple changes occur with progression of endocrine resistance in this model with AKT activation contributing to E_2_ insensitivity and loss of ERα(Ser118) phosphorylation being associated with full resistance. Cell cycle regulation is modified in endocrine-resistant breast cancer cells, and seliciclib is effective in both endocrine-sensitive and resistant diseases.

## Introduction

Endocrine therapy is a major treatment modality for estrogen receptor alpha (ERα)-positive breast cancer. However, despite initial sensitivity, most tumors eventually recur with acquired anti-estrogen resistance [1–5]. Multiple mechanisms have been described for the acquisition of resistance. These can differ dependently on whether tumors are treated with anti-estrogens or aromatase inhibitors [1–5]. In many instances, endocrine resistance is associated with enhanced dependency on human epidermal growth factor receptor (HER)/erbB receptor signaling where increased expression of either epidermal growth factor receptor [HER1/epidermal growth factor receptor (EGFR)/erbB1] or HER2/erbB2 can be associated with a poor response to tamoxifen [6–8].

Cancer cell line models are widely used to study and identify potential mechanisms of sensitivity and resistance. The ERα-positive MCF7 breast cancer cell line is both estrogen-sensitive and responsive to anti-estrogens such as tamoxifen and fulvestrant (Faslodex; ICI 182,780). Variant cell lines have been developed which are not only relatively estrogen-insensitive (MCF7/LCC1) [9] but have acquired resistance to anti-estrogens such as fulvestrant (LCC9) [10]. LCC1 cells were established from an MCF7 implant (MCF7/MIII) that was grown under low estrogen levels analogous to conditions produced by aromatase inhibitor treatment within an immunodeficient mouse. These cells were able to grow in the absence of estrogen but still responded to a lesser degree to estrogen and anti-estrogen treatment [9]. The LCC9 cell line was derived from LCC1 cells after treatment with fulvestrant and these cells are fully estrogen and anti-estrogen resistant [10].

Signaling activation via AKT represents a major pathway by which cells respond to growth factors and where activation and overexpression have been linked to endocrine resistance. The AKT protein kinases consist of three isoforms; AKT1, AKT2, and AKT3, and each isoform has been proposed to have a distinct biological role in mammary tumor progression [11–13]. Tamoxifen-resistant MCF7 cells have been shown to express increased P-AKT levels suggesting this pathway contributes to endocrine resistance [14]. Furthermore, activation of the phosphatidylinositol-3-kinase (PI3K)/AKT/mechanistic target of rapamycin (mTOR) pathway in ERα-positive breast cancers has been associated with relapse and death in patients treated with tamoxifen, supporting *in vitro* evidence that AKT mediates tamoxifen resistance [15].

Estrogen activates its receptor via phosphorylation at several key sites including serine 118 and serine 167, and these are critical to gene transcription [16]. Estrogen initiates a highly regulated series of cellular events to promote cell cycle progression and growth [17]. Increased expression of *MYC* is an early and pivotal response [18, 19]. MYC induces expression of key promoters of cell cycle progression including cyclin D1 [20, 21], cyclin E, and cyclin-dependent kinase 4 (CDK4) [22, 23], and is also implicated in the down-regulation of the CDK inhibitors p21 and p27 [24, 25]. The point at which the actions of MYC converge lies in the G1/S- transition phase of the cell cycle. Transition through this phase is governed by the cyclin E/CDK2 complex which, by hyperphosphorylation of the retinoblastoma protein (Rb) to generate hyperphosphorylated retinoblastoma protein (ppRb), uncouples and activates the early 2 factor (E2F) transcription factor, allowing transcription of genes required for cells to pass into the S-phase of the cell cycle [26]. In endocrine responsive breast cancer, anti-estrogen treatment with, e.g., tamoxifen or fulvestrant leads to down-regulation of MYC and induction of cell cycle arrest [27, 28]. However, with the onset of estrogen-independence and resistance to anti-estrogen therapies, these cell cycle effects of anti-estrogens are frequently lost. Indeed, constitutive expression of *MYC* is associated with resistance to anti-estrogen treatments [29], and MCF7 breast cancer cells which have acquired estrogen independence through long-term maintenance in estrogen-depleted media exhibit up-regulation of MYC and ERα [30]. In this situation, one potential therapeutic strategy is to bypass ERα signaling and directly target the cell cycle using a CDK inhibitor to inhibit cell growth.

Seliciclib (*R*-roscovitine, CYC202) is a potent CDK inhibitor that acts by competing with ATP for the ATP-binding site in the catalytic subunit of CDKs [31]. Seliciclib induces apoptosis and whilst functional p53 does not appear to be essential [32], several studies have indicated greater potency of seliciclib in cells with wild type as opposed to mutant p53 [33, 34]. Seliciclib has also been reported to induce cellular accumulation of *p53* [34, 35] and subsequent increase in *p21* [36] and p53 apoptosis-inducing protein 1 (*p53AIP1*) [33] expression. In MCF7 cells, seliciclib has been shown to promote phosphorylation of the Ser46 residue of p53, thereby increasing its stability, inducing the expression of *p53AIP1*, and subsequently increasing apoptosis [37]. While CDK2 may be its primary target, seliciclib can also inhibit other CDKs including CDK1, CDK5, CDK7, and CDK9 [38]. CDK7 does not have a direct role in regulating the transition of cells through stages of the cell cycle but rather serves to activate other CDKs by phosphorylation of the T-loop Thr160 residue, an event that is essential for their kinase activation. By targeting CDK7, seliciclib may effectively inhibit a number of other CDKs and indirectly block the cell cycle [38]. Through inhibiting CDK2/cyclin E, seliciclib has been shown to decrease the phosphorylation and ubiquitin-dependent degradation of p27, an inhibitor of CDK2 and CDK4. The subsequent accumulation of p27 can lead to cell cycle arrest in G1 [39]. Seliciclib-mediated reduction in the p53-regulating mouse double minute 2 (MDM2) [36] may also be a result of CDK7/CDK9 inhibition. It is likely that the growth inhibitory effects of seliciclib result from a combination of these multiple mechanisms [40]. Seliciclib has been shown to reverse intrinsic or acquired resistance to the aromatase inhibitor letrozole in breast cancer cells where low molecular weight cyclin E (LMW-E) is present [41]. Nair et al. [42] have demonstrated *in vitro* and *in vivo* activity of seliciclib in several MCF7 resistant models which individually have acquired resistance to tamoxifen, letrozole or have growth factor signaling cross-talk, and previous studies have investigated on the use of seliciclib in combination with other anti-tumor agents [38, 43].

In the present study, we investigated these signaling pathways in these resistant cell lines in order to determine further insight to the changes that can occur after endocrine treatment within breast cancer cells. We identified modified AKT signaling and reduced activation of ERα phosphorylation. We observed altered cell cycle control in the endocrine-resistant models and investigated the effects of seliciclib against these cell lines.

## Materials and methods

### Cell culture

MCF7 cells were grown in Dulbecco’s modified eagle medium (DMEM) with phenol-red, supplemented with 10% fetal calf serum (FCS), penicillin (100 units/mL), streptomycin (100 μg/mL) and 2 mmol/L glutamine. LCC1 and LCC9 cells (kindly provided by Prof. Robert Clarke, Vincent T. Lombardi Cancer Research Center, Georgetown University Medical School, Washington, D. C., USA) [9, 10] were maintained in phenol-red free containing DMEM supplemented with 5% dextran activated double charcoal stripped FCS (DCSS) + additives. MDA-MB-231 cells were obtained from American Type Culture Collection (ATCC) and were grown in the same medium as MCF7 cells. All cell lines were maintained in a humidified atmosphere at 37°C and 5% CO_2_ and confirmed mycoplasma free.

### Cell growth assays

Cells were counted using a Beckman Z2 Coulter counter. Log-phase cells were seeded into 24-well tissue culture plates (at cell densities optimized between 2–5 × 10^4^ cells mL^−1^) and treated with 5% DCSS phenol red-free DMEM containing 17β-estradiol (E_2_, 1 nmol/L), transforming growth factor alpha (TGFα, 1 nmol/L) or heregulin-beta (HRGβ, 1 nmol/L). Groups of wells were trypsinized on days 0, 3, 5, and 7, and cells were counted.

### Sulphorhodamine B assay

Cells were seeded into 96-well plates in a humidified atmosphere of 5% CO_2_/95% O_2_ at 37°C and cell number was determined using the sulphorhodamine B (SRB) assay [44]. MCF7 cells were seeded in 5% DCSS phenol red-free DMEM 48 h prior to treatment while resistant cell lines were seeded 24 h prior to treatment. Cells were then treated as indicated and the plates were incubated. The experiment was stopped at the times specified by the addition of 50 μL/well 25% trichloroacetic acid, 1 h, 4°C. Plates were then washed in water and when dry, 50 μL/well 4% SRB was added for 30 min at room temperature. Plates were washed in 1% acetic acid, left to dry and 150 μL/well 1.5 mol/L tris was added. After 1 h, the optical density of each plate at 540 nm was obtained using a Perkin Elmer plate reader. All ligands and reagents were obtained from Sigma-Aldrich unless otherwise stated. Seliciclib was obtained from Cyclacel Pharmaceuticals, Inc, Dundee and pertuzumab was kindly provided by Roche Diagnostics, Penzberg.

### Cell cycle and annexin V assays

Cells grown in DMEM/FCS were treated with either 1 nmol/L E_2_, TGFα or HRGβ for 72 h or in studies with seliciclib were treated with 20 μmol/L seliciclib for 24 h (with 0.1 nmol/L E_2 _). For cell cycle analysis, cells were resuspended in 100 μL of citrate buffer and stored at –20°C. Cells were subjected to flow cytometric DNA analysis using a FACSCalibre™ flow cytometer (Beckton Dickinson) as described previously [45]. Apoptosis was measured using the TACS annexin V-FITC kit (R&D Systems).

### Western analysis

Cells were treated as indicated with E_2_, TGFα, HRGβ or seliciclib in DMEM media containing 5% DCSS. Cell lysates were prepared as previously described [46]. Lysates were electrophoretically resolved on 10% sodium dodecyl sulfate-polyacrylamide gel electrophoresis (SDS-PAGE, with the exception of anti-human Rb where a 6% gel was used) and transferred to immobilon-P membranes. After transfer, membranes were probed overnight at 4°C with the appropriate primary antibody. All antibodies used were from Cell Signaling Technology, except for anti-human Rb (BD Pharmingen); anti-total ERα (Santa Cruz Biotechnology), anti-actin (Oncogene Research Products), anti-p53 (Oncogene), anti-CDK2 (Upstate Biotechnology, Inc); anti-tubulin (Abcam) and were used at 1:1,000 with the exception of actin (1:120,000). Immunoreactive bands were detected using enhanced chemiluminescent reagents (Roche) and hyperfilm enhanced chemiluminescence (ECL) film (Amersham^TM^). Integrated optical density (IOD) absorbance values were obtained by densitometric analysis using a gel scanner and analyzed by “Labworks^TM^” gel analysis software (UVP Life Sciences).

### Immunoprecipitation

For immunoprecipitation experiments, cells were lysed as described above and a volume of lysate containing 100 μg of protein was agitated overnight at 4°C with 1–10 μL of relevant antibody. Following overnight incubation, protein-G-agarose beads were washed in lysis buffer and 50 μL of the bead slurry was added to the lysate/antibody mix. The samples were then incubated for 3 h as before. Beads were washed three times in ice-cold lysis buffer. The lysate/antibody/bead slurry mixture was centrifuged for 2 min at 2,000 rpm (4°C) at the end of the three-hour incubation and the supernatant was collected using a syringe. Lysis buffer (minus protease inhibitors, 500 μL) was then added to the solution followed by another spin at 2,000 for 2 min. The supernatant was collected again, and the wash was repeated twice more as described. Loading buffer (20 μL) was added to the bead solution and the sample was heated at 95°C for 5 min. Samples were then centrifuged at 13,000 rpm for 1 min and the supernatant was removed for loading onto a polyacrylamide gel for western blot analysis.

### Small interfering RNA

AKT small interfering RNAs (siRNAs; 2.5 μL) were diluted in 250 μL of Opti-MEM in 6-well plates. Lipofectamine RNAiMAX (Invitrogen, 7.5 μL) was then added to each well, mixed gently and incubated for 10–20 min at room temperature. Cells were harvested as previously described and all cell lines were diluted to a concentration of 1.7 × 10^5^ cells/mL in 5% DCSS DMEM (-phenol red). The cell dilution (1.5 mL) was added to the RNAi-lipofectamine complexes and incubated for 48 h before RNA collection. For protein extraction plates, they were incubated for 72 h. siRNAs were used at a concentration of 50 nmol/L from a stock solution of 20 nmol/L. siRNA sequences were as follows: AKT1: 5’ACCTGACCAAGATGACAG; AKT2: 5’AAGTGGGTCCGCTGGT; AKT3: 5’AGGAGGTACAAGCTTTTTA (Applied Biosystems, UK); negative control siRNA (Upstate Biotechnology, Inc, M-003401).

### RNA extraction and reverse transcription polymerase chain reaction

Extraction of total RNA from whole cells was performed using tri-reagent (Sigma, Poole, Dorset) as per manufacturer instructions. RNA concentration was measured using a spectrophotometer. QuantiTect SYBR Green reverse transcription polymerase chain reaction (RT-PCR) kit (Quiagen Cat. #204243) was used according to the manufacturer’s instructions for one-step RT-PCR in a total of 15 μL reaction volumes including 10 μmol/L each primer and 40 ng RNA. The RotorGene PCR cycle conditions were: reverse transcription step 50°C for 30 min, Taq activation step 95°C for 15 min, PCR 40 cycles of 94°C for 15 s, 57°C for 30 s, and 72°C for 30 s. After a final extension of 72°C for 60 s, a PCR product melt curve was performed 60°–99°C. The following primer pairs were used:

β-actin: GATGGAGCCGCCGATCCACACGG, CTACGTCGCCCTGGACTTCGAGC
AKT1: ACCAGGTATTTTGATGAGGAGTTA, CGCTGTCCACACACTCCAT
AKT2: ATGCTGGCCGAGTAGGAGAA, GCCCAGTCCATCACAATC
AKT3: AGGACCGCACACGTTTCTAT, TTCTGGAGTGCCACAGAATG
MYC: TTCGGGTAGTGGAAAACCAG, AGCAGCTCGAATTTCTTCCA
Cyclin A1: ACCCCAAGAGTGGAGTTGTG, GGAAGGCATTTTCTGATCCA
Cyclin D1: AACTACCTGGACCGCTTCCT, CCACTTGAGCTTGTTCACCA
Cyclin E1: CAGATTGCAGAGCTGTTGGA, TCCCCGTCTCCCTTATAACC
CDK1: TTTTCAGAGCTTTGGGCACT, CCATTTTGCCAGAAATTCGT
CDK2: AAATTCATGGATGCCTCTGC, CAGGGACTCCAAAAGCTCTG
CDK4: GGGCAAAATCTTTGACCTGA, GAAAGGCAGAGATTCGCTTG
p21: GAGCGATGGAACTTCGACTT, CAGGTCCACATGGTCTTCCT
p27: ACCCCTAGAGGGCAAGTACG, ATCAGTCTTTGGGTCCACCA
p16: ACCCCGCTTTCGTAGTTTTC, CGTGAGTGCTCACTCCAGAA
p19: GTCATGATGTTTGGCAGCAC, CTGCCAGATGGATTGGAAGT
p53: CAAGGCCTCATTCAGCTCTC, GCGCACAGAGGAAGAGAATC
MDM2: CCAGGCTTTCATCAAAGGAA, GGTGGGAGTGATCAAAAGGA
p53AIP1: CTCTCCCCAGAAGCTCACAC, CTGGGACAGGAGGAACAAAA
p53 up-regulated modulator of apoptosis (PUMA): CTGTGAATCCTGTGCTCTGC, CTCCCTCTTCCGAGATTTCC

### p53 knockdown by stable expression of *p53* siRNA in MCF7 cells

The vector pSUPER.gfp+neo (VEC-pBS-0006) into which was inserted a sequence encoding an effective siRNA targeting p53 isolated from the vector pSUPER.p53 (VEC-p53.0001) was used. Sequence was checked by direct sequencing through the siRNA expression cassette and validated by lipofectin mediated transfection of the relinearized pSUPER.p53.gfp+neo vector into the MCF7 cell line which resulted in effective p53 knockdown in these cells. The p53 knockdown status in these cells in culture was maintained by growth in 400 ug/mL geneticin. These cell lines are referred to as MCF7-p53-KD.

### Statistics

Groups were compared by analysis of variance (ANOVA) followed by the Tukey-Kramer multiple comparison test.

## Results

### Reduced growth response to estrogen is reflected in modified HER-activated growth in resistant models

**Figure 1.**
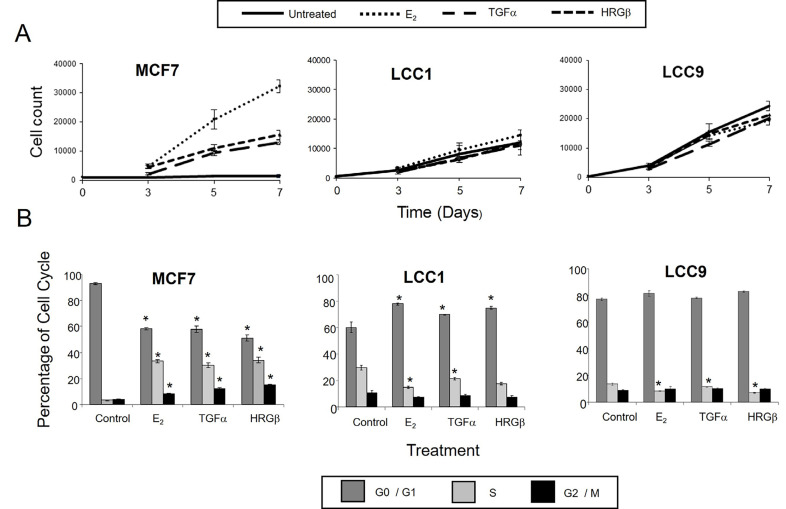
Effects of E_2_, TGFα, and HRGβ on cell growth and cell cycle distribution in MCF7, LCC1, and LCC9 cell lines. A. Cell growth response to E_2_, TGFα and HRGβ. Cells were grown for 24 h prior to treatment, then treated with 1 nmol/L growth factors or hormone. Cells were counted on the days indicated. Data shown are mean values of quadruplicates and are representative of three independent experiments. B. Cell cycle analysis after 72 h treatment with growth factors or hormone. Results are representative of three independent experiments. * statistically significant changes between control and treatment (*P* < 0.05)

The LCC1 and LCC9 cell lines represent models of increasing degrees of estrogen insensitivity relative to wild-type estrogen-sensitive MCF7 cells. We first assessed whether the reduced response to estrogen was also associated with changed sensitivity to the HER activating ligands TGFα and HRGβ. These growth factors activate signaling via HER dimerization, predominantly HER1 homodimers or HER1/HER2 heterodimers for TGFα and HER3/erbB2 for HRGβ. The effects of E_2_ on growth and cell cycle distribution were compared with those of TGFα and HRGβ. MCF7 cells grew poorly in DCSS FCS but were growth stimulated by treatment (1 nmol/L) with E_2_, TGFα, and HRGβ (Figure 1A). In contrast, the LCC1 and LCC9 cell lines proliferated in the absence of ligand stimulation. LCC1 cells demonstrated a minimal growth response to E_2_, and were insensitive to TGFα and HRGβ compared to wild type MCF7 cells, while LCC9 cells were insensitive to all treatments (Figure 1A).

**Figure 2.**
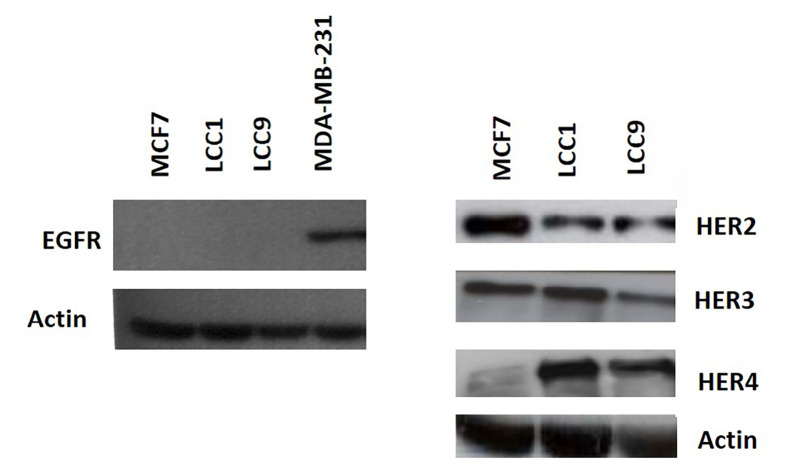
Expression of the HER receptors in the cell lines. Western analysis of the 3 cell lines and MDA-MB-231 (positive control for EGFR) as described in “Materials and methods”

Consistent with the growth effects in MCF7 cells, cell cycle analysis demonstrated that 1 nmol/L E_2_, TGFα, and HRGβ increased the percentage of cells in S- and G2/M phases (Figure 1B). In contrast, LCC1 and LCC9 cells had a much higher percentage of cells initially in S-phase in the absence of hormone or growth factors and this percentage was not increased by their addition (Figure 1B). These data indicate collateral resistance between E_2_ and the growth factors in these progressively resistant cell lines. Protein expression levels of HER2 were lower in LCC1 and LCC9 cells relative to MCF7 cells while HER4 expression was increased. HER3 expression was similar across the 3 cell lines. EGFR protein was below the limit of detection for the cell lines but shown to be present in MDA-MB-231 cells (as a positive control) (Figure 2).

### P-AKT is increased in the resistant cell lines

**Figure 3.**
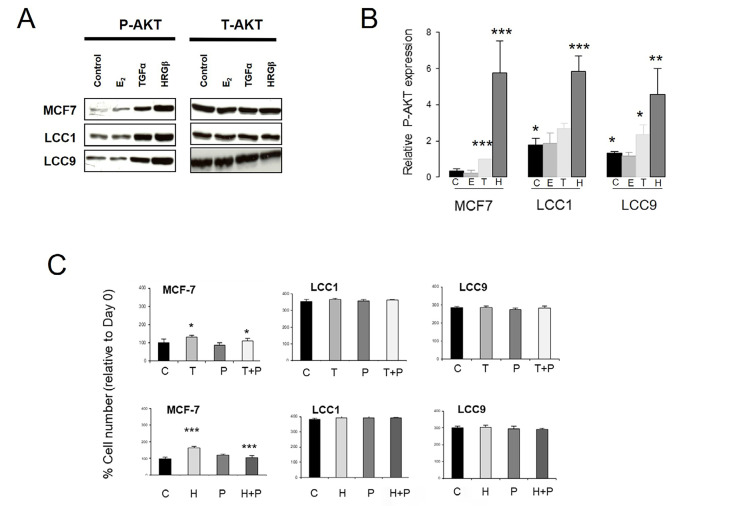
Comparison of the effect of E_2_, TGFα and HRGβ stimulation on expression levels of P-AKT(Ser473) in the wild-type and resistant cell lines. A. Western blot of P-AKT [P-AKT(Ser473)] and T-AKT levels in cell lines after 15 min treatment with E_2_, TGFα or HRGβ (1 nmol/L). B. Histogram of relative P-AKT expression levels (relative to T-AKT) in the cell lines after C, E, T or H. ANOVA test, *n* = 3. C. Effect of P in the absence or presence of growth factor T or H on growth over 72 h in the cell lines. Cell number was assessed by SRB assay as described in “Materials and methods”. C: untreated control; E: 1 nmol/L E_2_; H: 1 nmol/L HRGβ; P: 100 nmol/L pertuzumab; T: 1 nmol/L TGFα; * *P* < 0.05; ** *P* < 0.01; *** *P* < 0.001

We next investigated whether the AKT pathway was differentially activated in the sensitive and resistant cell lines (Figure 3). Expression of P-AKT(Ser473) was significantly increased in both LCC1 and LCC9 cells relative to MCF7 cells in the absence of ligands, with levels of total AKT (T-AKT) unchanged (Figure 3A, 3B). Addition of either TGFα or HRGβ increased P-AKT expression in all three lines while E_2_ had no effect (Figure 3A, 3B).

The anti-HER2 antibody pertuzumab could reverse the growth stimulations produced by either TGFα or HRGβ in MCF7 cells as previously reported [47], however it was ineffective in the LCC1 and LCC9 cell lines (Figure 3C), again indicating a changed growth dependence on these pathways in the resistant cell lines.

### Differential expression of *AKT* isoforms in the resistant cell lines

**Figure 4.**
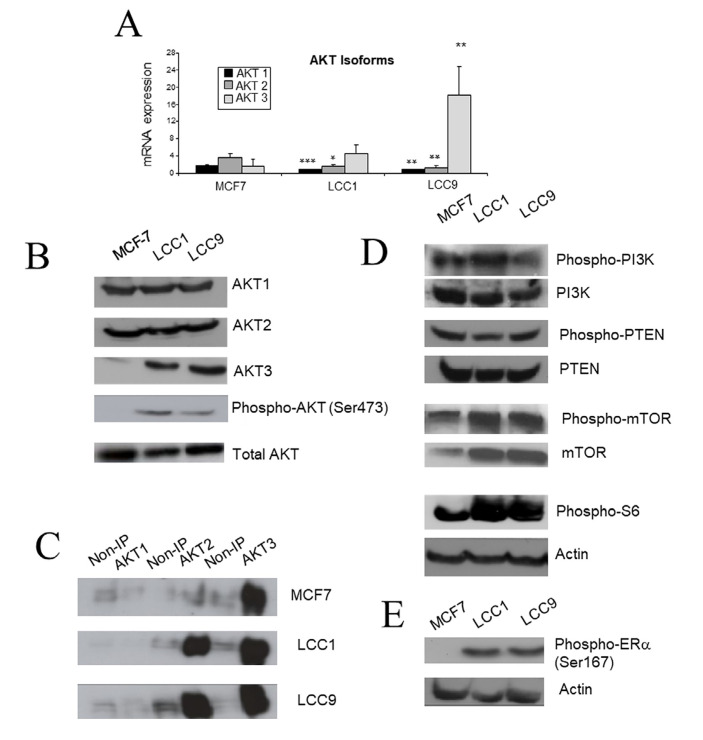
*AKT* expression and associated signaling in the cell lines. A. Expression of *AKT* isoform mRNA measured by RT-PCR. Each column represents mean of quadruplicate values relative to actin expression. Error bar = standard deviation (SD). Statistical comparison of resistant line with MCF7; * *P* < 0.05; ** *P* < 0.01; *** *P* < 0.001 (ANOVA). B. Western blot of AKT isoforms, P-AKT(Ser743) and total AKT in the cell lines. C. P-AKT isoforms. Samples were immunoprecipitated with AKT isoform specific antibodies and then probed for P-AKT. The non-immunoprecipitated (IP) control was incubated with immunoglobulin G (IgG) rather than isoform specific antibody. D. Western blots of upstream (PI3K, PTEN) and downstream (mTOR, S6) signaling molecules of AKT in the cell lines. E. Western blot of phospho-ERα(Ser167) in the cell lines

Since AKT pathway activation was increased in the resistant cell lines, we next investigated whether there was differential expression of individual isoforms of *AKT* in these cell lines. Expressions of *AKT1* and *AKT2* mRNA were significantly lower in LCC1 and LCC9 cells in comparison to parental MCF7 cells (Figure 4A). In contrast, *AKT3* mRNA expression was elevated in LCC1 and LCC9 cells. Assessment of protein expression by western analysis indicated similar expression of AKT1 and AKT2 across the three cell lines, but increased expression of AKT3 in LCC1 and LCC9 cells relative to MCF7 cells (Figure 4B). Despite these differences in individual isoforms, the total level of the three isoforms combined was similar in the three cell lines (Figure 4B).

To assess phosphorylation levels of specific *AKT* isoforms, immunoprecipitation was performed using AKT isoform specific antibodies. The immunoprecipitates were then probed with anti-P-AKT(Ser473) antibody to reveal which isoforms were activated in each of the cell lines. In the three cell lines, AKT3 is highly phosphorylated (Figure 4C). The level of AKT3 phosphorylation progressively increases in LCC1 and LCC9 in comparison to MCF7, and levels of AKT2 phosphorylation are also increased (Figure 4C). Activation of signaling downstream of AKT was then investigated. In LCC1 and LCC9 cells, both phospho-mTOR and phospho-S6 expression were increased, consistent with enhanced AKT activation, although total mTOR is already increased in LCC1 and LCC9 cells (Figure 4D). Furthermore, ERα was phosphorylated at its Ser167 residue consistent with the pathway proposed by Campbell et al. [48] (Figure 4E). We have previously reported ERα expression to be increased in LCC1 and LCC9 cells [49].

**Figure 5.**
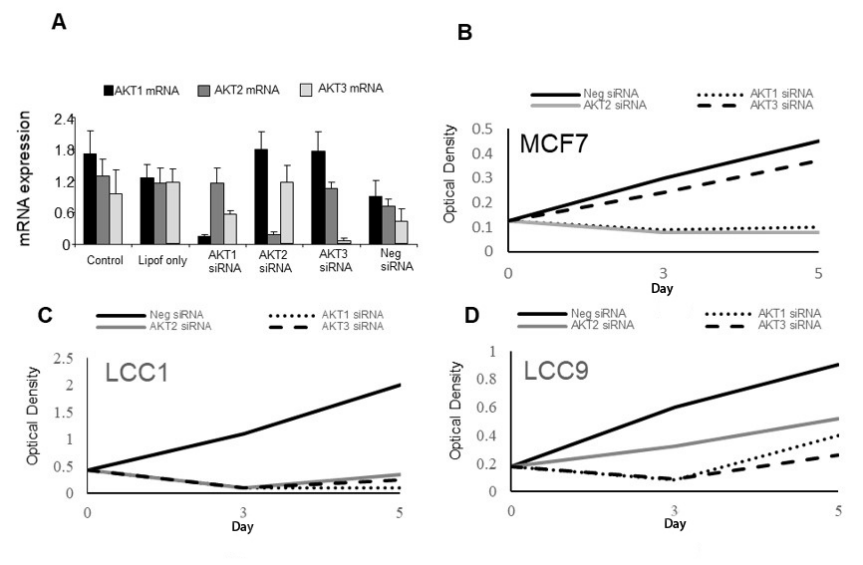
Effects of *AKT* siRNAs on the expression and growth of the cell lines. A. Effect of *AKT* siRNAs on specific *AKT* isoform expression. B–D. Effect of *AKT* siRNAs (50 nmol/L) on proliferation of (B) MCF7 cells, (C) LCC1 cells, and (D) LCC9 cells. Each point represents the mean of 6 values. Neg: negative control

To test the effects of inhibiting individual *AKT* isoform expression, an siRNA approach was adopted. Specificity of mRNA knockdown was first demonstrated using LCC9 cells (Figure 5A) and this cell line was used as these cells express all 3 isoforms (Figure 4B). Cell growth of parental MCF7 cells were sensitive to *AKT1* and *AKT2* specific siRNAs as cell number is reduced while *AKT3* siRNA had little effect (Figure 5B). LCC1 cells were sensitive to all three *AKT* siRNAs as they all reduced growth while LCC9 cells were also inhibited by all three siRNAs but less so with *AKT2* siRNA (Figure 5C, 5D).

### Comparison of the effect of E_2_ and TGFα treatment on phospho-ERα(Ser 118) levels in the resistant cell lines

**Figure 6.**
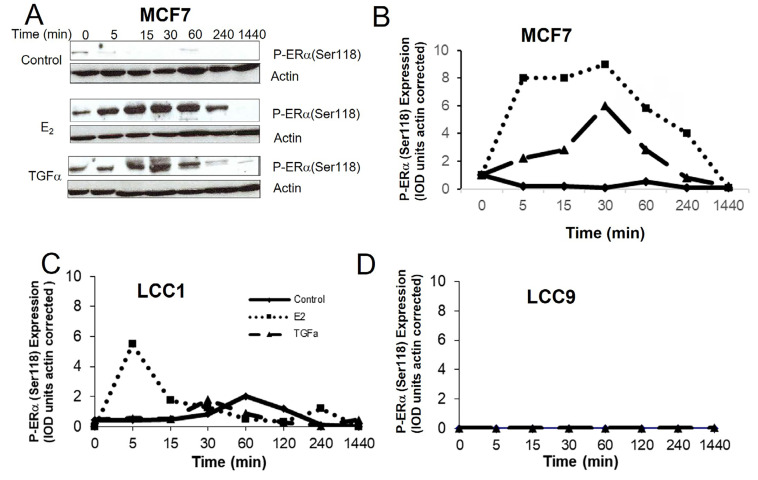
The effects of E_2_ and TGFα on the expression of P-ERα(Ser118) in the cell lines. A. Western blot example of P-ERα(Ser118) expression for MCF7 cells treated with E_2_ or TGFα. B–D. Time courses of the effect of E_2_ or TGFα on P-ERα(Ser118) expression for (B) MCF7 cells, (C) LCC1 cells, or (D) LCC9 cells. Legend for B–D is shown in panel C

Estrogen binding to ERα results in phosphorylation of Ser118 of the receptor and this can also be achieved by growth factor signaling resulting in ligand-independent activation [50]. The effects of E_2_ and TGFα on phospho-ERα(Ser118) expression in the cell lines were compared over a time-course (Figure 6). In MCF7 cells, both E_2_ and TGFα markedly increased P-ERα(Ser118) expression over several hours (Figure 6A, 6B). In LCC1 cells, E_2_ increased P-ERα(Ser118) expression after 5 min for a short period of time but this rapidly reversed while TGFα had no effect on expression in this cell line (Figure 6C). Neither E_2_ nor TGFα increased P-ERα(Ser118) expression in LCC9 cells (Figure 6D).

### Loss of growth stimulation by estrogen coincides with multiple changes in the pathway that links ERα to cell cycle regulation

**Figure 7.**
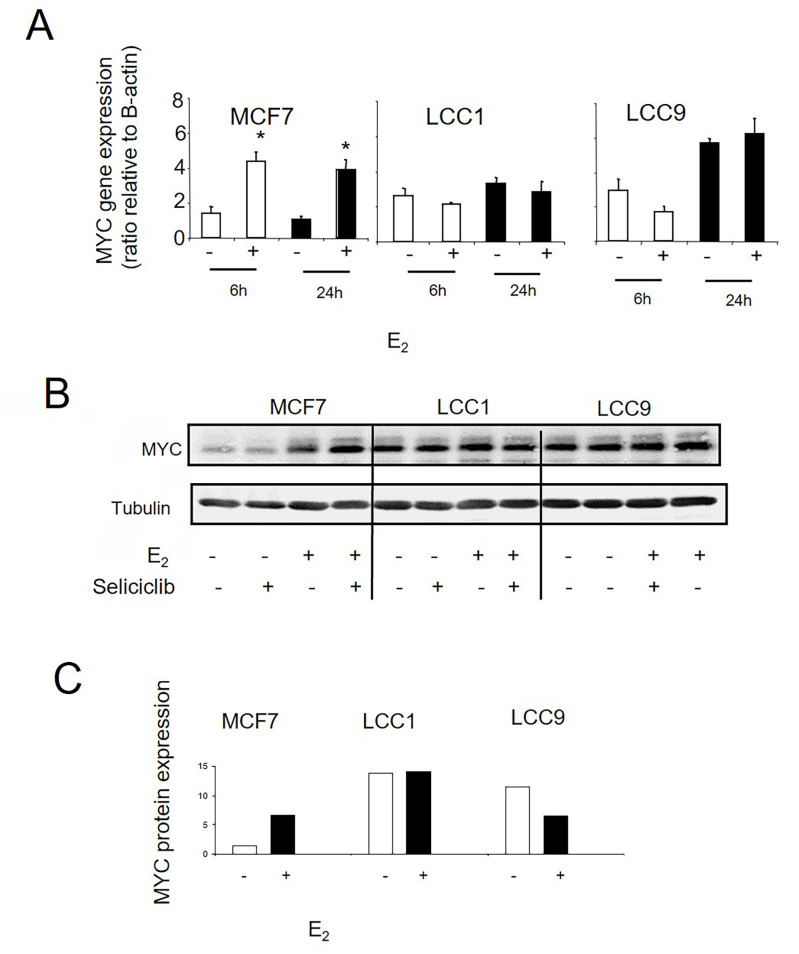
A. *MYC* mRNA expression as determined by RT-PCR in cell lines grown +/– E_2_ (0.1 nmol/L). Data shown are expressed as the ratio of MYC: *β-actin* expression. Mean values +/– SDs are shown. Values were then normalized against the MCF7 control. Groups were compared by ANOVA followed by the Tukey-Kramer multiple comparison test. * *P* < 0.05. B. Western blot of MYC protein expression in cell lines grown +/– E_2_ (0.1 nmol/L) and +/– seliciclib. C. Data shown are expressed as a ratio of MYC: tubulin expression. +: modulator shown present; −: modulator shown absent

We next investigated whether stepwise acquisition of increasing endocrine resistance is accompanied by a progressive loss of control of the estrogen-mediated cell cycle drive. Treatment with E_2_ leads to increased *MYC* mRNA expression in MCF7 cells after 6 h which is maintained at 24 h (Figure 7A). By comparison, levels of MYC in LCC1 and LCC9 cells are already elevated relative to MCF7 in the absence of E_2_ stimulation and are not induced further by E_2_ (Figure 7A). These differences in *MYC* expression are also reflected at the protein level (Figure 7B, 7C).

**Figure 8.**
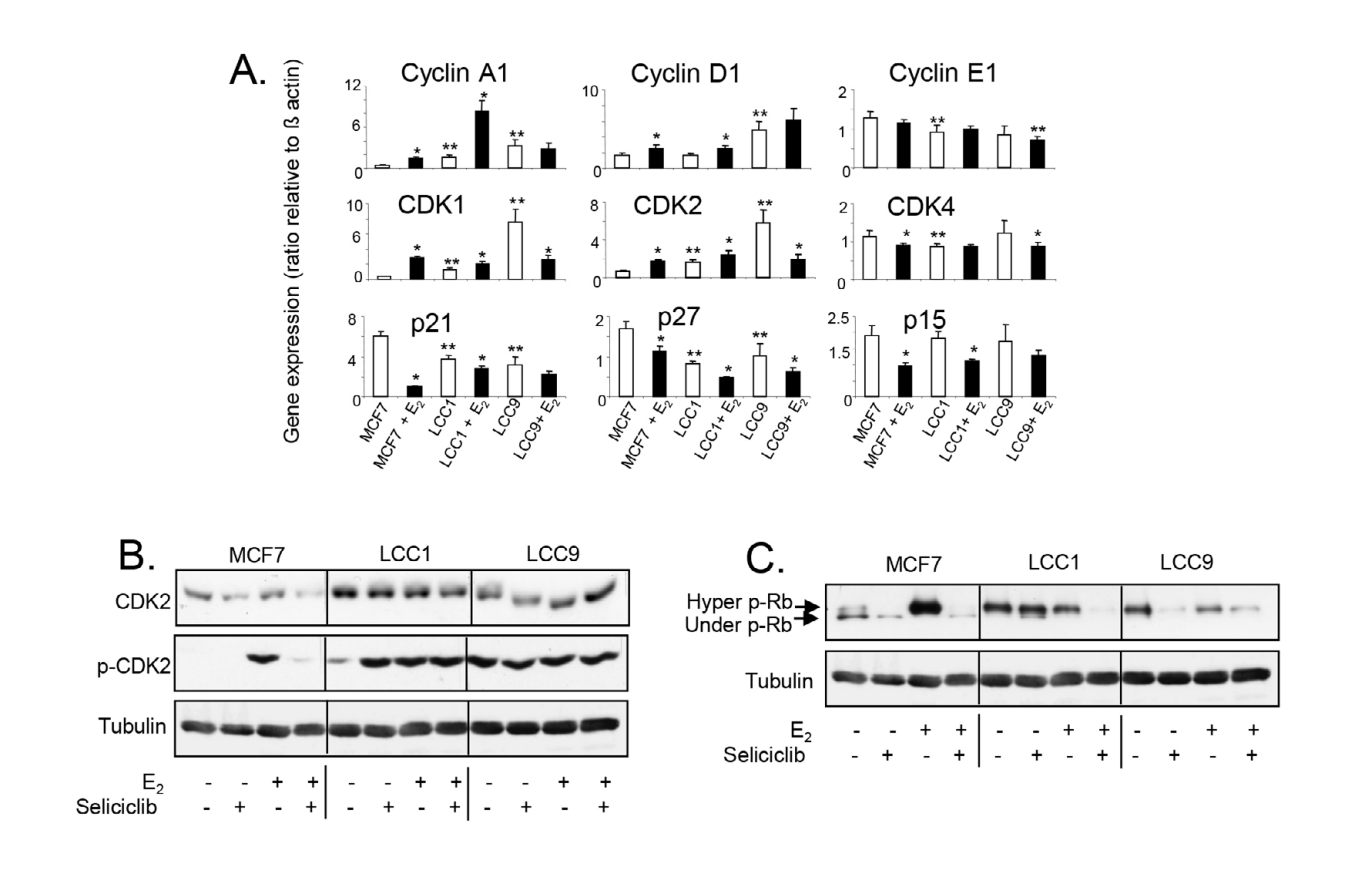
A. Expression of cell cycle regulatory gene mRNA levels in cell lines grown +/– E_2_ (0.1 nmol/L) for 24 h. Data shown are expressed as the ratio of gene expression relative to that of β-actin. Mean of quadruplicate values is shown. Statistically significant values are indicated by either * (comparison with E_2_-treatment, *P* < 0.05) or ** (comparison with MCF7 in absence of E_2_, *P* < 0.05). Groups were compared by ANOVA followed by the Tukey-Kramer multiple comparison test. B. Western blot showing total and phosphorylated *CDK2* expression in cell lines exposed to E_2_ (0.1 nmol/L) +/– seliciclib (20 μmol/L) for 24 h. C. Western blot showing Rb phosphorylation in cell lines exposed to E_2_ (0.1 nmol/L) +/– seliciclib (20 μmol/L) for 24 h. +: modulator shown present; −: modulator shown absent

Altered basal expression of *MYC* in LCC1 and LCC9 and E_2_ induction of *MYC* in MCF7 are reflected in progressive changes in expression and regulation of a number of cell cycle regulators (Figure 8A). Levels of cyclin A1 mRNA are elevated in LCC1 and LCC9 relative to MCF7 cells and are strongly increased in both MCF7 and LCC1 by E_2_ but not in LCC9 cells consistent with growth response. Expression of *cyclin D1* (but not *cyclin E*) is elevated in LCC9 but not LCC1 cells and E_2_ increases expression of *cyclin D1* in both E_2_-growth responsive MCF7 and LCC1 cells. Expression of the *CDK1* and *CDK2* (but not *CDK4*) are increased on exposure to E_2_ in MCF7 and LCC1 cells but decreased in LCC9 which has elevated basal expression. Expression of the cell cycle inhibitors *p21*, *p27*, and *p15* are reduced in the MCF7 and LCC1 cell lines on exposure to E_2_. Expression of *p21* and *p27* are highest in untreated MCF7 cells and the most marked repression of *p21* by E_2_ is seen in this cell line.

Elevated expression of CDK2 protein in LCC1 and LCC9 cells was confirmed by western blotting (Figure 8B). Although E_2_ does not markedly affect its expression in MCF7 cells, a profound difference was seen in its activation (phosphorylation of Thr160) when cells were exposed to E_2_. LCC1 cells have elevated basal activation of CDK2 (compared to MCF7 cells) but this was further increased by E_2_, while the elevated level of phosphorylated CDK2 seen in LCC9 was not further increased when cells were exposed to E_2_. Seliciclib reduced both the levels of total CDK2 and phosphorylated CDK2 in MCF7 cells. While seliciclib increased CDK2 phosphorylation in LCC1 cells in the absence of E_2_, it had little effect on total CDK2 in these cells and no effects in LCC9 cells.

The target for active cyclin E/CDK2 is Rb and therefore its hyper-phosphorylation status was evaluated after treatment with E_2_ and/or seliciclib (Figure 8C). Both LCC1 and LCC9 cells showed increased basal levels of ppRb compared to MCF7 and exposure to E_2_ resulted in decreased hyperphosphorylation in these cells (Figure 8C). Conversely, in MCF7 cells, exposure to estrogen leads to a very significant increase in ppRb. Seliciclib reduced hyperphosphorylation in both the presence and absence of E_2_. Collectively, these data indicate that in MCF7 cells, estrogen modulates cell cycle regulators. In LCC1 cells some elements of estrogen-control are lost, e.g., MYC induction and an increase in ppRb whilst some estrogen responsiveness is retained (phosphorylated CDK2, cyclin A1), consistent with a residual degree of estrogen cell growth drive. The LCC9 cell line shows no estrogen-mediated growth response and, consistent with this, estrogen has no impact on these signaling events.

### Seliciclib inhibits cell growth, blocks the cell cycle and induces apoptosis in both endocrine sensitive and resistant cell lines

**Figure 9.**
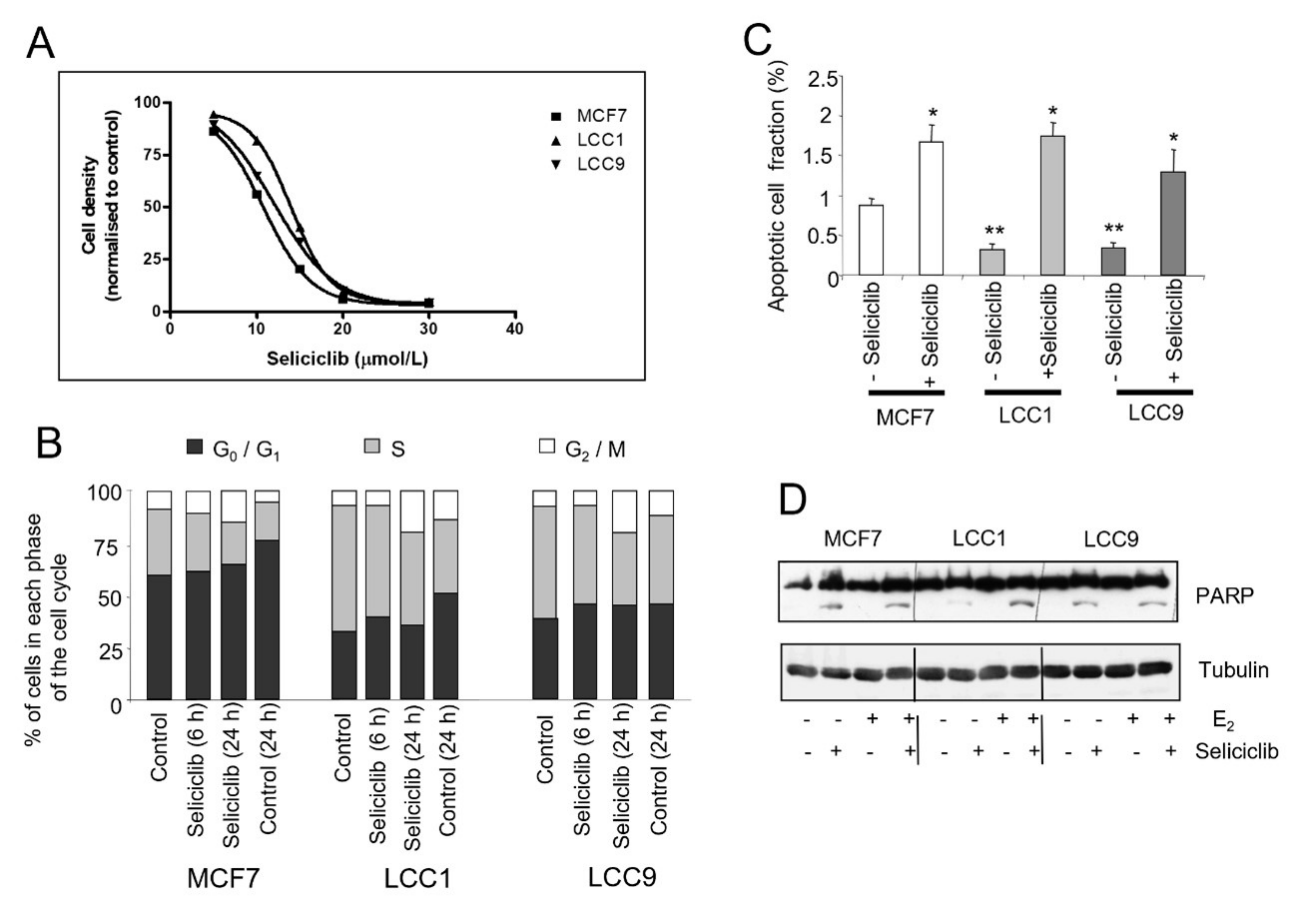
Effects of seliclib on cell growth, cell cycle and apoptosis in the cell lines. A. Inhibition of cell growth in cell lines as determined by seliciclib. The cell number is shown after 3 days treatment and the day 0 value is indicated for comparison. B. Cell cycle distribution of cell lines grown in E_2_ (0.1 nmol/L) +/– seliciclib (20 μmol/L) for 24 h. Groups were compared by ANOVA followed by the Tukey-Kramer multiple comparison test. * *P* < 0.05 (comparison with same cell line control); ** *P* < 0.05 (comparison with MCF7 control). C. Proportion of apoptotic cells as determined by annexin V assay in E_2_ (0.1 nmol/L) +/– seliciclib (20 μmol/L) for 24 h. Mean values +/– SDs are shown. D. Induction of apoptosis in cell lines exposed to seliciclib (20 μmol/L) +/– E_2_ (0.1 nmol/L) for 24 h. PARP cleavage is represented by the lower row of bands (cleaved PARP) with full-length PARP above. PARP: polyadenosine-diphosphate-ribose polymerase; +: modulator shown present; −: modulator shown absent

These cell lines were next treated with seliciclib; all three cell lines were inhibited to a similar degree (EC_50_ of 11 μmol/L for MCF7; 14 μmol/L for LCC1 and 12.5 μmol/L for LCC9) (Figure 9A). Cell cycle studies indicated that all three cell lines showed a similar percentage of cells accumulating in G2/M on exposure to seliciclib (Figure 9B).

MCF7 cells have the highest basal level of apoptosis but seliciclib increased apoptosis to a comparable extent in all three cell lines as determined by measurement of annexin V (Figure 9C) and PARP cleavage (Figure 9D).

### Role of p53 in the growth inhibition by seliciclib

**Figure 10.**
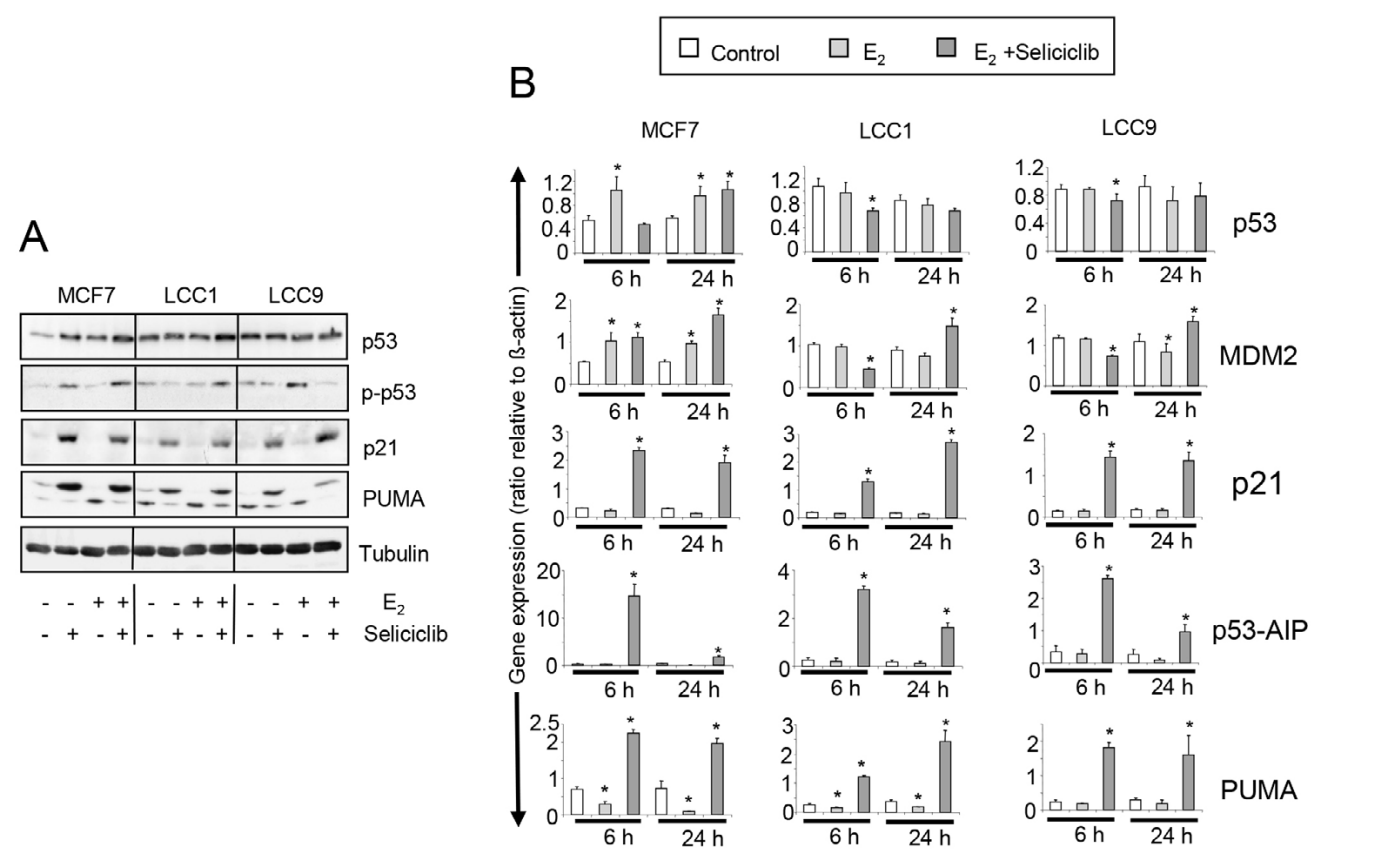
A. Western blots showing total expression and phosphorylation of *p53*, along with *p21* and PUMA expression in cell lines grown +/– E_2_ (0.1 nmol/L), +/– seliciclib (20 μmol/L). Lysates were harvested after 24 h. B. Expression of *p53*, *MDM2* and downstream regulators of apoptosis in cell lines grown +/– E_2_ (0.1 nmol/L), +/– seliciclib (20 μmol/L). Data shown are the expression of the named gene relative to that of *β-actin*. Mean values +/– SDs are shown. Groups were compared by ANOVA followed by the Tukey-Kramer multiple comparison test. * *P* < 0.05

The expression and activation profiles of *p53* are altered in both LCC1 and LCC9 cells compared with MCF7 cells (Figure 10A, 10B). Low basal *p53* expression in MCF7 is increased by estrogen and seliciclib to a level comparable with that found in unstimulated LCC1 and LCC9 cells. No increase in *p53* expression was seen on exposure of these latter cell lines to either seliciclib or estrogen. Seliciclib increased phosphorylation at the Ser46 residue of p53 in MCF7 cells and this is augmented by estrogen (Figure 10A). Phosphorylation of this residue is important in regulating the ability of p53 to induce apoptosis [37]. In LCC1 cells, seliciclib alone does not increase phosphorylation of Ser46 but is able to do this in the presence of estrogen. In LCC9 cells, seliciclib does not increase the level of Ser46 phosphorylation. Expression of *p21*, *PUMA*, and *p53AIP1* mRNAs are strongly increased in MCF7, LCC1 and LCC9 cells by seliciclib (Figure 10B), but induction of *p53AIP1* mRNA after 6 h is much greater in MCF7 than in the other cell lines (Figure 10B). This is consistent with earlier observations that *p53AIP1* expression is specifically induced when p53 is phosphorylated on Ser46 in response to seliciclib [51].

**Figure 11.**
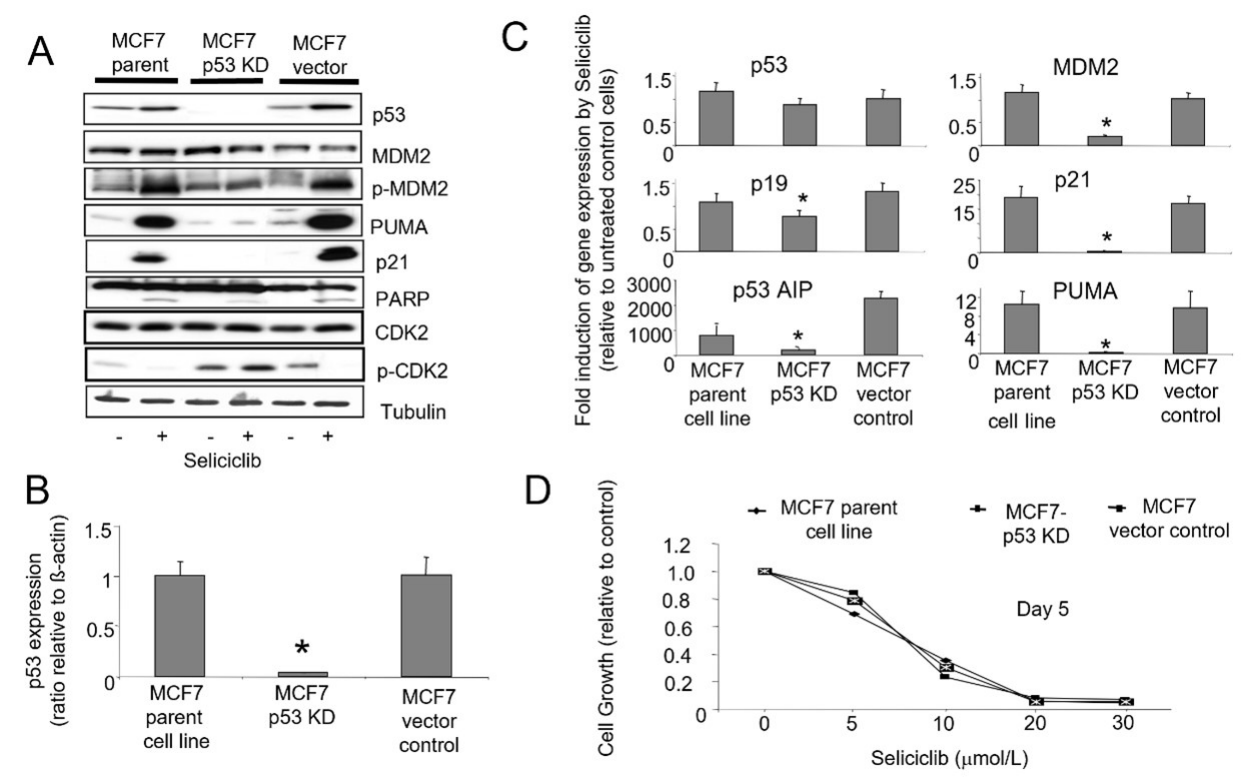
A. Western blots showing expression and phosphorylation of *p53*, *MDM2* and *CDK2* together with *p21* and *PUMA* in MCF7-parent and MCF7-p53-KD cell lines in +/– seliciclib (20 μmol/L) for 24 h. Cleaved PARP expression illustrates the levels of apoptosis under these conditions. B. Expression of *p53* mRNA in cell lines. Data shown are the ratio of expression of *p53* relative to that of *β-actin*. C. Effect of seliciclib on the expression of *p53* and associated genes in cell lines. The data shown is a ratio of expression of the named gene in seliciclib-treated relative to untreated cells (after correction of target mRNA relative to actin). * comparison with MCF7 parent cell line control, *P* < 0.05. D. Growth inhibitory effect of seliciclib in cell lines as determined using the SRB growth assay. +: modulator shown present; −: modulator shown absent

To further evaluate the role of p53 in mediating the cellular effects of seliciclib, we stably expressed *p53* targeting siRNA in MCF7 cells resulting in knockdown of p53. We compared clones with p53 knockdown with vector controls and wild type cells (Figure 11). We observed that induction of p21, PUMA and p53AIP1 by seliciclib is p53-dependent, and clones lacking *p53* expression show corresponding reduction in induction of these known p53 transcriptional targets. Loss of p53 also results in a reduced level of *MDM2* mRNA (Figure 11C) but does not affect the levels of protein expression (Figure 11A). In MCF7 parent and vector control cells, exposure to seliciclib results in increased phosphorylation of MDM2 Ser166, an event normally mediated by AKT which allows MDM2 to gain nuclear entry and thereby regulate levels of p53 [52]. When *p53* expression is reduced by stable siRNA expression in MCF7 cells, we see loss of this activation. Together, these observations imply a reduction in turnover of MDM2 following siRNA mediated p53 knockdown. Despite these effects, the degree of seliciclib-induced apoptosis as demonstrated by PARP cleavage in these clones does appear independent of the degree of siRNA mediated p53 knockdown (Figure 11A). Cell growth experiments using a range of seliciclib concentrations shows that p53 status does not alter the overall growth inhibition of MCF7 derived clones by seliciclib (Figure 11D).

## Discussion

We report on signaling changes in two widely used ERα-positive endocrine-resistant breast cancer cell lines. We explored the growth factor driven AKT pathways that is known to contribute to estrogen-independent activation of ERα and we investigated components of cell cycle control regulated by estrogen that might differ in resistant cells.

Growth of these endocrine-resistant cell lines was not only insensitive to estrogen but was also insensitive to TGFα and HRGβ unlike the parent MCF7 cell line. One frequently observed mechanism of endocrine resistance in ERα-positive breast cancer is via increased expression of *EGFR* or *HER2* [6–8] and this can lead to increased sensitivity to HER inhibitors [6–8]. In the LCC1 and LCC9 cell lines, *HER* expression levels were reduced rather than increased and this was associated with resistance (rather than sensitivity) to the HER2 inhibitor pertuzumab.

Previously published studies using MCF7 cells made endocrine-resistant through a variety of routes have reported that P-AKT [14, 48, 53] is frequently activated in resistant cells. AKT activation was identified in the LCC1 and LCC9 cell lines and this has been previously observed in MCF7 cell lines made resistant to tamoxifen [14, 54]. Furthermore, it is also reported that transfection of AKT into MCF7 cells renders them resistant to tamoxifen providing direct support for a role for AKT overexpression in resistance [48]. AKT3 protein expression was increased in the LCC1 and LCC9 cell lines relative to wild-type MCF7 and immunoprecipitation suggested that both AKT2 and AKT3 had strong constitutive signaling. siRNA reduction suggested though that all 3 isoforms contributed to growth response in the resistant cell lines. Increased expression of *AKT3* has previously been shown to produce estrogen-independence and resistance to fulvestrant in MCF7 cells consistent with AKT3 having a role here in the LCC cell lines [55]. While upstream *pPI3K* and *pPTEN* expression were unchanged in both resistant cell lines, downstream signaling through mTOR, S6 and ERα were increased consistent with AKT activation. AKT activation can positively regulate the cell cycle through its impact on cyclin D1, Rb, p21 and p27 among other regulators [13] and these are evaluated below.

Activation of ERα by E_2_ is associated with phosphorylation of serine residues at positions 118 and 167 [16]. Growth factor signaling can also activate Ser118 phosphorylation of ERα leading to estrogen-independent activation [50]. Chromatin immunoprecipitation analysis has demonstrated that phospho-ERα(Ser118) is associated with the promoters of estrogen-regulated genes in MCF7 breast cancer cells 30 min following estrogen treatment [56]. As observed in other studies, E_2_ and TGFα activated ERα(Ser118) phosphorylation in MCF7 cells. In contrast, the effect of E_2_ in LCC1 cells was transient and TGFα had no effect while neither E_2_ nor TGFα activated ERα(Ser118) phosphorylation in LCC9 cells. One reported estrogen-independent cell line, derived by Staka et al. [57], the MCF7X model has a number of signaling alterations in common with the LCC models. This cell line is also growth-insensitive to growth factor receptor stimulation, and it possesses elevated P-AKT expression compared to the parental cell line. However, it differs in those levels of P-ERα(Ser118) were constitutively elevated in the MCF7X cell line relative to the parent line but this was not seen for the LCC cell lines.

**Figure 12.**
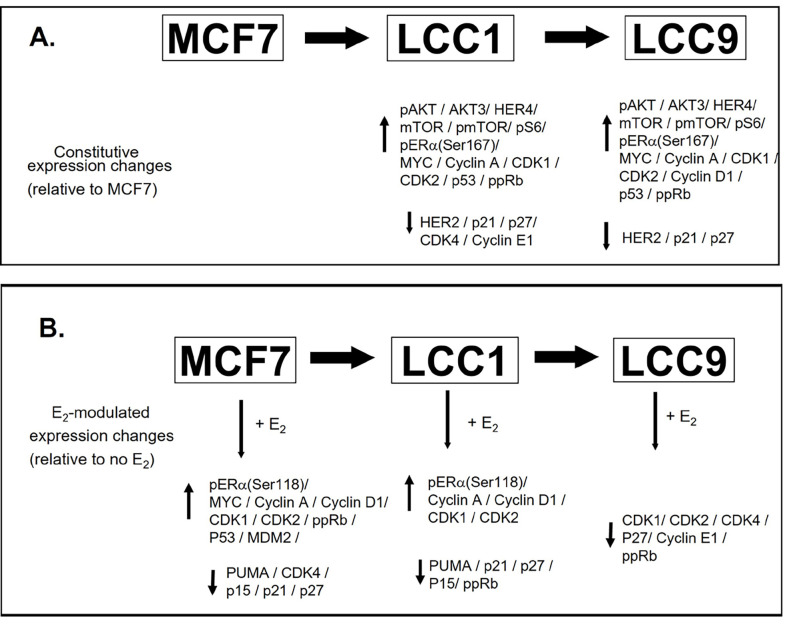
Summary of the constitutive expression changes between the cell lines and estrogen-modulated expression changes. A. Changes between the cell lines. B. Estrogen-modulated expression changes. ↑: increased expression; ↓: reduced expression

The series of cellular events whereby ERα drives the cell cycle machinery is well defined, and de-regulation of this machinery is frequently found in endocrine-resistant disease [58]. The high constitutive expression of *MYC* observed in LCC1 and LCC9 cells may contribute to anti-estrogen resistance and this is consistent with published data that link elevated *MYC* expression with anti-estrogen resistance [59]. Expression of the *CDKs* and *cyclins* was increased in MCF7 cells as a result of estrogen treatment and a similar pattern is observed in LCC1 cells consistent with activation of estrogen signaling. However, in LCC9 cells, most of these estrogen-modulated changes are lost. The constitutive expression differences between the cell lines and the estrogen-modulated expression changes described in this study are summarized (Figure 12).

While clinical studies have focused predominantly on the use of CDK4/6 inhibitors to treat endocrine-resistant breast cancer [60, 61], and use of CDK2/1 inhibitors has also been considered [42, 62]. The study reported by Nair et al. [42] demonstrated the ability of seliciclib to inhibit growth of several types of endocrine-resistant cell line models with associated inhibitory effects on CDK2 activation. In our study of MCF7 cells, estrogen promoted CDK2 activation, and this was blocked by seliciclib. LCC1 cells have an increased basal level of CDK2 phosphorylation which was further enhanced by exposure to estrogen but increased rather than blocked by seliciclib. Interestingly, in MCF7-p53-knockdown clones, there is an increased basal level of activated CDK2 which is not reduced by seliciclib unlike parental MCF7 or clones with near wild type *p53* expression levels. p53 has previously been shown to be a target for phosphorylation by CDK7 [63] and there is evidence that the CDK-activating kinase (CAK, CDK7/cyclinH/MAT-1 complex) mediated activating phosphorylation of CDK2 is regulated by p53 such that increasing cell levels of wild type p53 lead to a reduction in CDK2 phosphorylation [64]. In the resistant models, LCC1 and LCC9 cells expressed elevated levels of p53 compared to MCF7 cells although this was increased by estrogen to a comparable level. In MCF7 cells, activation of p53 by Ser46 phosphorylation coincided with increased expression of *p21*, *PUMA* and *p53AIP1* but not in LCC1 and LCC9 cells. In all three cell lines, seliciclib gave similar levels of growth inhibition, cell cycle arrest and induction of apoptosis. Using MCF7 cells stably expressing *p53* siRNA, we show that *p53* is required for seliciclib mediated induction of p21, PUMA and p53AIP1 but not for apoptosis. The multiple actions of seliciclib may be a clinical strength. The present results suggest that in acquiring endocrine-independence, LCC1 and LCC9 cells have altered drive of the cell cycle machinery and that seliciclib may inhibit growth via different targets. It appears however that this has not affected the efficacy of this agent and in all cell lines treatment produces similar cell cycle arrest, apoptosis, and growth inhibition. These observations provide further support for consideration of use of this type of agent in endocrine-resistant cancers.

In conclusion, this report characterizes further a range of features found in the endocrine-resistant breast cancer cells. While certain modifications of signaling are observed in other resistant models, the unique combination of features found here adds further insight into the broad width of aberrations that can occur in endocrine-resistant cells.

## Abbreviations

ANOVA: analysis of variance
CDK1: cyclin-dependent kinase 1
DCSS: double charcoal stripped fetal calf serum
DMEM: Dulbecco’s modified eagle medium
E_2_: 17β-estradiol
EGFR: epidermal growth factor receptor
ERα: estrogen receptor alpha
FCS: fetal calf serum
HER: human epidermal growth factor receptor
HRGβ: heregulin-beta
IOD: integrated optical density
MDM2: mouse double minute 2
mTOR: mechanistic target of rapamycin
p53AIP1: p53 apoptosis-inducing protein 1
PARP: polyadenosine-diphosphate-ribose polymerase
PI3K: phosphatidylinositol 3-kinase
ppRb: hyperphosphorylated retinoblastoma protein
PUMA: p53 up-regulated modulator of apoptosis
Rb: retinoblastoma protein
RT-PCR: reverse transcription polymerase chain reaction
SD: standard deviation
siRNA: small interfering RNA
SRB: sulphorhodamine B
TGFα: transforming growth factor alpha

## Declarations

### Copyright

© The Author(s) 2022.
